# *catena*-Poly[[diiodidocadmium]-μ-[4,4′-(2,3,5,6-tetra­methyl-1,4-phenyl­ene)bis­(methyl­ene)]bis­(3,5-dimethyl-1*H*-pyrazole)-κ^2^ *N* ^2^:*N* ^2′^]

**DOI:** 10.1107/S1600536811025797

**Published:** 2011-07-06

**Authors:** Yuanyuan Zhou, Zhaoyang Wang, Guang Yang, Seik Weng Ng

**Affiliations:** a Department of Chemistry, Zhengzhou University, Zhengzhou 450001, People’s Republic of China; b Department of Chemistry, University of Malaya, 50603 Kuala Lumpur, Malaysia; c Chemistry Department, Faculty of Science, King Abdulaziz University, PO Box 80203 Jeddah, Saudi Arabia

## Abstract

The heterocylic ligand of the polymeric title compound, [CdI_2_(C_22_H_30_N_4_)], links two adjacent CdI_2_ units, forming a chain running parallel to [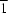
01]. The Cd^II^ atom is located on a twofold rotation axis and shows a distorted tetra­hedral CdI_2_N_2_ coordination. The mid-point of the benzene ring of the ligand lies on a center of inversion. There are no classical hydrogen-bonding inter­actions present.

## Related literature

For the synthesis of the ligand, see: Trofimenko (1970 ▶).
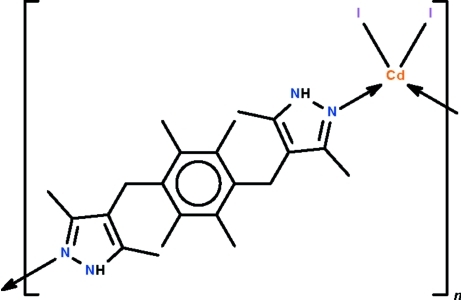


## Experimental

### 

#### Crystal data

[CdI_2_(C_22_H_30_N_4_)]
*M* *_r_* = 716.70
Monoclinic, 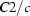

*a* = 22.118 (8) Å
*b* = 6.840 (2) Å
*c* = 17.057 (6) Å
β = 93.407 (5)°
*V* = 2575.8 (16) Å^3^
*Z* = 4
Mo *K*α radiation
μ = 3.26 mm^−1^
*T* = 293 K
0.20 × 0.20 × 0.20 mm

#### Data collection

Rigaku Saturn 724 CCD diffractometer
Absorption correction: multi-scan (*CrystalClear*; Rigaku, 2006 ▶) *T*_min_ = 0.763, *T*_max_ = 1.000
14901 measured reflections
2951 independent reflections
2589 reflections with *I* > 2σ(*I*)
*R*_int_ = 0.041

#### Refinement

*R*[*F* ^2^ > 2σ(*F* ^2^)] = 0.054
*wR*(*F* ^2^) = 0.134
*S* = 1.18
2951 reflections
137 parameters
H-atom parameters constrained
Δρ_max_ = 1.12 e Å^−3^
Δρ_min_ = −0.74 e Å^−3^

### 

Data collection: *CrystalClear* (Rigaku, 2006 ▶); cell refinement: *CrystalClear*; data reduction: *CrystalClear*; program(s) used to solve structure: *SHELXS97* (Sheldrick, 2008 ▶); program(s) used to refine structure: *SHELXL97* (Sheldrick, 2008 ▶); molecular graphics: *X-SEED* (Barbour, 2001 ▶); software used to prepare material for publication: *publCIF* (Westrip, 2010 ▶).

## Supplementary Material

Crystal structure: contains datablock(s) I, global. DOI: 10.1107/S1600536811025797/wm2507sup1.cif

Structure factors: contains datablock(s) I. DOI: 10.1107/S1600536811025797/wm2507Isup2.hkl

Additional supplementary materials:  crystallographic information; 3D view; checkCIF report

## Figures and Tables

**Fig. 1.**
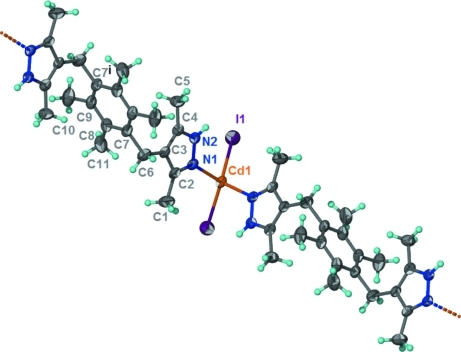
Figure 1. Thermal ellipsoid plot of a portion of the polymeric chain structure of CdI2(C22H30N4). Ellipsoids are drawn at the 50% probability level. [Symmetry code: i) -x+1/2, -y+5/2, -z.]

## supplementary crystallographic information

### Comment

The heterocyclic ligand is a new member of the class of geminal bis-(1-pyrazol-1-yl)alkanes developed fourty years ago by Trofimenko, who also investigated its coordination abilities (Trofimenko, 1970). However, no crystal structures of adducts of the parent 4,4'-(1,4-phenylene)bis(methylene)bis(3,5-dimethyl-1*H*-pyrazole) ligand have been reported to date.

The heterocylic ligand of the polymeric title compound CdI_2_(C_22_H_30_N_4_) (Fig. 1) links two adjacent CdI_2_ units to form a chain running parallel to [101]. The Cd^II^ atom shows a distorted tetrahedral CdI_2_N_2_ coordination. The 1*H*-pyrazole H atom is not involved in hydrogen bonding interactions, presumably due to the presence of the bulky methyl group and the CdI_2_ unit in its vicinity.

### Experimental

A solution of cadmium diiodide (7.3 mg, 0.02 mmol) in methanol (2 ml) was added to a solution of 4,4'-(2,3,5,6-tetramethyl-1,4-phenylene)bis(methylene)bis(3,5-dimethyl-1*H*-pyrazole) (3.5 mg, 0.01 mmol) in ethanol (2 ml). The solution was allowed to evaporate for several days to afford colorless crystals in 80% yield. Calc. for C_22_H_30_N_4_CdI_2_: C 36.87, H 4.22, N 7.82%. Found: C 36.71, H 4.25, N 7.69%.

### Refinement

H atoms were placed in calculated positions [C—H 0.93–0.98 Å, N–H 0.88 Å; *U*(H) = 1.2–1.5*U*_eq_(C,*N*)]. The highest peak in the final difference Fourier map is in the vicinity (1.35 Å) of H10B.

### Crystal data

**Table:**

[CdI_2_(C_22_H_30_N_4_)]	*F*(000) = 1376
*M**_r_* = 716.70	*D*_x_ = 1.848 Mg m^−^^3^
Monoclinic, *C*2/*c*	Mo *K*α radiation, λ = 0.71073 Å
Hall symbol: -C 2yc	Cell parameters from 3092 reflections
*a* = 22.118 (8) Å	θ = 2.1–30.6°
*b* = 6.840 (2) Å	µ = 3.26 mm^−^^1^
*c* = 17.057 (6) Å	*T* = 293 K
β = 93.407 (5)°	Cuboid, colorless
*V* = 2575.8 (16) Å^3^	0.20 × 0.20 × 0.20 mm
*Z* = 4	

### Data collection

**Table:**

Rigaku Saturn 724 CCD diffractometer	2951 independent reflections
Radiation source: fine-focus sealed tube	2589 reflections with *I* > 2σ(*I*)
graphite	*R*_int_ = 0.041
φ and ω scans	θ_max_ = 27.5°, θ_min_ = 2.4°
Absorption correction: multi-scan (*CrystalClear*; Rigaku, 2006)	*h* = −28→28
*T*_min_ = 0.763, *T*_max_ = 1.000	*k* = −8→8
14901 measured reflections	*l* = −22→21

### Refinement

**Table:**

Refinement on *F*^2^	Secondary atom site location: difference Fourier map
Least-squares matrix: full	Hydrogen site location: inferred from neighbouring sites
*R*[*F*^2^ > 2σ(*F*^2^)] = 0.054	H-atom parameters constrained
*wR*(*F*^2^) = 0.134	*w* = 1/[σ^2^(*F*_o_^2^) + (0.0555*P*)^2^ + 7.4182*P*] where *P* = (*F*_o_^2^ + 2*F*_c_^2^)/3
*S* = 1.18	(Δ/σ)_max_ = 0.001
2951 reflections	Δρ_max_ = 1.12 e Å^−^^3^
137 parameters	Δρ_min_ = −0.74 e Å^−^^3^
0 restraints	Extinction correction: *SHELXL97* (Sheldrick, 2008), Fc^*^=kFc[1+0.001xFc^2^λ^3^/sin(2θ)]^-1/4^
Primary atom site location: structure-invariant direct methods	Extinction coefficient: 0.00092 (15)

### Special details

**Table:**

Geometry. All e.s.d.'s (except the e.s.d. in the dihedral angle between two l.s. planes) are estimated using the full covariance matrix. The cell e.s.d.'s are taken into account individually in the estimation of e.s.d.'s in distances, angles and torsion angles; correlations between e.s.d.'s in cell parameters are only used when they are defined by crystal symmetry. An approximate (isotropic) treatment of cell e.s.d.'s is used for estimating e.s.d.'s involving l.s. planes.

### Fractional atomic coordinates and isotropic or equivalent isotropic displacement parameters (Å^2^)

**Table:**

	*x*	*y*	*z*	*U*_iso_*/*U*_eq_	
I1	0.53637 (2)	0.41499 (7)	0.12116 (3)	0.0692 (2)	
Cd1	0.5000	0.60117 (8)	0.2500	0.0424 (2)	
N1	0.43399 (19)	0.8146 (7)	0.1908 (2)	0.0438 (10)	
N2	0.44190 (19)	0.8589 (6)	0.1144 (2)	0.0421 (10)	
H2	0.4695	0.8045	0.0863	0.051*	
C1	0.3667 (4)	0.9231 (11)	0.2924 (4)	0.071 (2)	
H1A	0.3239	0.8970	0.2889	0.106*	
H1B	0.3741	1.0464	0.3181	0.106*	
H1C	0.3873	0.8213	0.3220	0.106*	
C2	0.3894 (2)	0.9304 (8)	0.2115 (3)	0.0433 (12)	
C3	0.3678 (2)	1.0496 (8)	0.1477 (3)	0.0409 (11)	
C4	0.4023 (2)	0.9968 (8)	0.0868 (3)	0.0421 (11)	
C5	0.4040 (3)	1.0599 (9)	0.0030 (3)	0.0554 (15)	
H5A	0.3636	1.0599	−0.0210	0.083*	
H5B	0.4287	0.9710	−0.0246	0.083*	
H5C	0.4206	1.1892	0.0009	0.083*	
C6	0.3180 (3)	1.1960 (10)	0.1503 (3)	0.0590 (16)	
H6A	0.3353	1.3203	0.1674	0.071*	
H6B	0.2905	1.1547	0.1893	0.071*	
C7	0.2817 (2)	1.2271 (8)	0.0728 (3)	0.0438 (12)	
C8	0.2379 (2)	1.0890 (7)	0.0473 (4)	0.0469 (13)	
C9	0.2061 (2)	1.1132 (8)	−0.0256 (3)	0.0460 (12)	
C10	0.1571 (3)	0.9684 (10)	−0.0503 (5)	0.0701 (19)	
H10A	0.1639	0.9195	−0.1018	0.105*	
H10B	0.1183	1.0318	−0.0512	0.105*	
H10C	0.1578	0.8617	−0.0137	0.105*	
C11	0.2249 (3)	0.9125 (9)	0.0990 (5)	0.0687 (19)	
H11A	0.2565	0.8998	0.1397	0.103*	
H11B	0.2232	0.7962	0.0675	0.103*	
H11C	0.1868	0.9310	0.1223	0.103*	

### Atomic displacement parameters (Å^2^)

**Table:**

	*U*^11^	*U*^22^	*U*^33^	*U*^12^	*U*^13^	*U*^23^
I1	0.0695 (3)	0.0714 (4)	0.0668 (3)	0.0130 (2)	0.0040 (2)	−0.0205 (2)
Cd1	0.0394 (3)	0.0439 (3)	0.0428 (3)	0.000	−0.0077 (2)	0.000
N1	0.039 (2)	0.053 (3)	0.038 (2)	0.007 (2)	−0.0068 (18)	−0.001 (2)
N2	0.037 (2)	0.048 (2)	0.041 (2)	0.0054 (19)	−0.0001 (18)	−0.0006 (19)
C1	0.084 (5)	0.087 (5)	0.040 (3)	0.029 (4)	0.004 (3)	−0.001 (3)
C2	0.043 (3)	0.048 (3)	0.037 (3)	0.003 (2)	−0.010 (2)	−0.005 (2)
C3	0.037 (3)	0.046 (3)	0.038 (2)	0.007 (2)	−0.008 (2)	−0.005 (2)
C4	0.044 (3)	0.039 (3)	0.042 (3)	0.003 (2)	−0.006 (2)	−0.001 (2)
C5	0.067 (4)	0.058 (3)	0.042 (3)	0.018 (3)	0.010 (3)	0.001 (3)
C6	0.060 (4)	0.072 (4)	0.043 (3)	0.025 (3)	−0.008 (3)	−0.011 (3)
C7	0.039 (3)	0.045 (3)	0.047 (3)	0.017 (2)	−0.007 (2)	−0.005 (2)
C8	0.040 (3)	0.039 (3)	0.062 (3)	0.007 (2)	−0.001 (2)	0.006 (2)
C9	0.036 (3)	0.042 (3)	0.060 (3)	0.006 (2)	−0.004 (2)	−0.009 (2)
C10	0.052 (4)	0.055 (4)	0.101 (5)	−0.002 (3)	−0.011 (4)	−0.016 (4)
C11	0.050 (4)	0.063 (4)	0.093 (5)	0.004 (3)	0.009 (3)	0.024 (4)

### Geometric parameters (Å, °)

**Table:**

I1—Cd1	2.7034 (8)	C5—H5B	0.9600
Cd1—N1^i^	2.260 (4)	C5—H5C	0.9600
Cd1—N1	2.260 (4)	C6—C7	1.520 (7)
Cd1—I1^i^	2.7034 (8)	C6—H6A	0.9700
N1—C2	1.329 (7)	C6—H6B	0.9700
N1—N2	1.358 (6)	C7—C9^ii^	1.392 (8)
N2—C4	1.353 (6)	C7—C8	1.403 (8)
N2—H2	0.8800	C8—C9	1.403 (8)
C1—C2	1.497 (8)	C8—C11	1.532 (8)
C1—H1A	0.9600	C9—C7^ii^	1.392 (8)
C1—H1B	0.9600	C9—C10	1.509 (8)
C1—H1C	0.9600	C10—H10A	0.9600
C2—C3	1.420 (7)	C10—H10B	0.9600
C3—C4	1.375 (7)	C10—H10C	0.9600
C3—C6	1.491 (7)	C11—H11A	0.9600
C4—C5	1.494 (7)	C11—H11B	0.9600
C5—H5A	0.9600	C11—H11C	0.9600
			
N1^i^—Cd1—N1	99.6 (2)	C4—C5—H5C	109.5
N1^i^—Cd1—I1	116.88 (12)	H5A—C5—H5C	109.5
N1—Cd1—I1	98.99 (11)	H5B—C5—H5C	109.5
N1^i^—Cd1—I1^i^	98.99 (11)	C3—C6—C7	114.9 (4)
N1—Cd1—I1^i^	116.88 (12)	C3—C6—H6A	108.5
I1—Cd1—I1^i^	123.80 (4)	C7—C6—H6A	108.5
C2—N1—N2	105.2 (4)	C3—C6—H6B	108.5
C2—N1—Cd1	137.3 (4)	C7—C6—H6B	108.5
N2—N1—Cd1	117.2 (3)	H6A—C6—H6B	107.5
C4—N2—N1	111.9 (4)	C9^ii^—C7—C8	120.3 (5)
C4—N2—H2	124.1	C9^ii^—C7—C6	120.1 (5)
N1—N2—H2	124.1	C8—C7—C6	119.6 (5)
C2—C1—H1A	109.5	C7—C8—C9	119.7 (5)
C2—C1—H1B	109.5	C7—C8—C11	120.2 (5)
H1A—C1—H1B	109.5	C9—C8—C11	120.1 (5)
C2—C1—H1C	109.5	C7^ii^—C9—C8	120.0 (5)
H1A—C1—H1C	109.5	C7^ii^—C9—C10	121.0 (5)
H1B—C1—H1C	109.5	C8—C9—C10	119.0 (5)
N1—C2—C3	111.1 (5)	C9—C10—H10A	109.5
N1—C2—C1	121.4 (5)	C9—C10—H10B	109.5
C3—C2—C1	127.5 (5)	H10A—C10—H10B	109.5
C4—C3—C2	104.6 (4)	C9—C10—H10C	109.5
C4—C3—C6	130.0 (5)	H10A—C10—H10C	109.5
C2—C3—C6	125.4 (5)	H10B—C10—H10C	109.5
N2—C4—C3	107.2 (4)	C8—C11—H11A	109.5
N2—C4—C5	118.8 (5)	C8—C11—H11B	109.5
C3—C4—C5	133.9 (5)	H11A—C11—H11B	109.5
C4—C5—H5A	109.5	C8—C11—H11C	109.5
C4—C5—H5B	109.5	H11A—C11—H11C	109.5
H5A—C5—H5B	109.5	H11B—C11—H11C	109.5
			
N1^i^—Cd1—N1—C2	−76.4 (5)	N1—N2—C4—C5	−178.8 (5)
I1—Cd1—N1—C2	164.2 (5)	C2—C3—C4—N2	−1.1 (6)
I1^i^—Cd1—N1—C2	28.8 (6)	C6—C3—C4—N2	179.4 (6)
N1^i^—Cd1—N1—N2	96.0 (4)	C2—C3—C4—C5	179.3 (6)
I1—Cd1—N1—N2	−23.4 (4)	C6—C3—C4—C5	−0.2 (11)
I1^i^—Cd1—N1—N2	−158.7 (3)	C4—C3—C6—C7	29.6 (9)
C2—N1—N2—C4	−1.4 (6)	C2—C3—C6—C7	−149.9 (5)
Cd1—N1—N2—C4	−176.1 (3)	C3—C6—C7—C9^ii^	−99.5 (7)
N2—N1—C2—C3	0.7 (6)	C3—C6—C7—C8	77.4 (7)
Cd1—N1—C2—C3	173.7 (4)	C9^ii^—C7—C8—C9	−0.5 (9)
N2—N1—C2—C1	179.2 (5)	C6—C7—C8—C9	−177.5 (5)
Cd1—N1—C2—C1	−7.7 (9)	C9^ii^—C7—C8—C11	179.5 (5)
N1—C2—C3—C4	0.3 (6)	C6—C7—C8—C11	2.6 (8)
C1—C2—C3—C4	−178.2 (6)	C7—C8—C9—C7^ii^	0.5 (9)
N1—C2—C3—C6	179.9 (5)	C11—C8—C9—C7^ii^	−179.5 (5)
C1—C2—C3—C6	1.4 (9)	C7—C8—C9—C10	−177.1 (5)
N1—N2—C4—C3	1.6 (6)	C11—C8—C9—C10	2.9 (8)

Symmetry codes: (i) −*x*+1, *y*, −*z*+1/2; (ii) −*x*+1/2, −*y*+5/2, −*z*.

## References

[bb1] Barbour, L. J. (2001). *J. Supramol. Chem.* **1**, 189–191.

[bb2] Rigaku (2006). *CrystalClear* Rigaku/MSC Inc., The Woodlands, Texas, USA.

[bb3] Sheldrick, G. M. (2008). *Acta Cryst.* A**64**, 112–122. 10.1107/S0108767307043930 18156677

[bb4] Trofimenko, S. (1970). *J. Am. Chem. Soc.* **92**, 5118–5126.

[bb5] Westrip, S. P. (2010). *J. Appl. Cryst.* **43**, 920–925.

